# Long-term use of opioids and neuropathic medication after diaphyseal humerus fractures with or without nerve injury: a nationwide register-based cohort study of 4,185 patients

**DOI:** 10.2340/17453674.2026.46808

**Published:** 2026-10-30

**Authors:** Erika NYMAN, Simone ANDERSSON, Raquel PEREZ, Malin ZIMMERMAN, Juan MERLO, Lars B DAHLIN

**Affiliations:** 1 Department of Orthopedic and Hand Surgery, Faculty of Medicine and Health, Örebro University, Örebro; 2 Clinical Department of Hand Surgery, Plastic Surgery and Burns, Region Östergötland, Linköping University Hospital, Linköping; 3 Department of Biomedical and Clinical Sciences, Linköping University, Linköping; 4 Department of Orthopedics, Kalmar Hospital, Kalmar; 5 Department of Translational Medicine – Hand Surgery, Lund University, Skåne University Hospital, Malmö; 6 Unit for Social Epidemiology, Department of Clinical Sciences (Malmö), Faculty of Medicine, Lund University, Malmö; 7 Department of Orthopedics, Helsingborg Hospital, Helsingborg; 8 Center for Primary Health Research, Region Skåne, Malmö, Sweden

## Abstract

**Background and purpose:**

Diaphyseal humerus fractures may lead to prolonged pain medication use. We aimed to study pain medication use after such fractures, focusing on nerve injury.

**Methods:**

Using Swedish register data (2007–2015), we identified individuals with diaphyseal humerus fractures. High use of opioids and neuropathic pain medication (≥ 60% of days covered/year; 2-month post-fracture washout) was evaluated in 5 groups: the general population (reference) and fractures treated nonoperatively or surgically, with and without nerve injury. Relative risks (RRs; 95% confidence intervals [CIs]; crude and adjusted [aRR] models; adjusted for age, sex, country of birth, income, and cohabitation status; and for previous high use of pain medication during the first year when analyzing the second year) were estimated in Cox proportional hazards regression models.

**Results:**

We identified 4,185 individuals with diaphyseal humerus fractures (3.7% with nerve injury; 23% surgically treated). In the first year, opioid use was higher among surgically treated fractures with nerve injury (aRR 17.6, CI 11.0–28.3) and without nerve injury (aRR 9.5, CI 8.2–10.9) than in the general population. In the second year, opioid use declined across most groups but remained elevated among nonoperatively treated fractures with nerve injury (aRR 8.3, CI 2.6–26.1). Neuropathic pain medication use increased in the first year, with no elevated use in the second. Adjustment did not change overall patterns. Women, older adults, lower-income individuals, and those living alone showed higher medication risk use.

**Conclusion:**

Patients with a diaphyseal humerus fracture were associated with a substantial burden of pain medication use, particularly for opioids during the first year post-injury in surgically treated fractures with nerve injury (RR 17.6) and a persistent risk in the second year among nonoperatively treated fractures with nerve injury (RR 8.3). Demographics and socioeconomic factors strongly relate to medication use, highlighting the need for careful early pain management and monitoring for prolonged medication use in exposed groups.

Diaphyseal humerus fractures account for about 1–3% of all fractures and show a bimodal age distribution, with 1 peak in the third decade, often linked to high-energy trauma, and a second peak in the eighth decade, usually caused by low-energy mechanisms, which may result in pain [1-3]. Nonoperative treatment using functional bracing has traditionally been the preferred management strategy for these fractures [4-6]. Surgical treatment is generally reserved for specific indications, including open fractures, vascular injuries, severe soft-tissue damage, multiple traumata, pathological fractures, manipulation-induced secondary radial nerve injury, or failure of fracture healing after nonoperative treatment [7-9]. Nerve injury is a well-recognized complication of diaphyseal humerus fractures, with the radial nerve most frequently affected and reported in approximately 8–16% of cases [1,8,10-12]. Although most radial nerve injuries recover spontaneously, with reported full recovery rates of 70–90% over 1–16 months [13,14], a subset of individuals experience persistent neurological deficits. Persistent nerve injury after upper limb trauma, in general, has been associated with substantial pain and functional impairment [15]. However, pain-related outcomes have rarely been examined at the population level in this context.

Despite the clinical relevance of pain management after diaphyseal humerus fractures, particularly in the presence of nerve injury, population-based evidence on long-term use of opioids and neuropathic pain medications remains limited. Moreover, treatment strategy and nerve injury status may be expected to influence pain trajectories and subsequent pharmacological management, yet few studies have examined these factors in relation to prolonged medication use. At the same time, demographic and socioeconomic characteristics are strongly associated with pain medication use in the general population, underscoring the need to distinguish fracture-related associations from underlying population differences.

Therefore, the aim of our nationwide register-based cohort study was to examine long-term use of opioids and neuropathic pain medication after diaphyseal humerus fractures. Specifically, we compared the risk of prolonged medication use among individuals in different fracture categories, defined by treatment strategy and the presence of nerve injury, with that of the general population.

## Methods

### Study design

We used linked data from several Swedish population registers, which contain individual-level information covering the entire Swedish population. The unique Swedish personal identity number was used and anonymized prior to analysis. Data was extracted from the National Patient Register (NPR; nationwide data on inpatient care since 1987 and specialized outpatient care since 2001) and the Swedish Prescribed Drug Register (SPDR) to obtain all registered diagnoses and surgical codes, as well as drug dispensations. NPR has virtually complete coverage of inpatient care and generally high diagnostic validity, with positive predictive values typically reported between 85% and 95% for many conditions, though validity varies by diagnosis. It tends to be higher for broader diagnostic categories than for highly specific ICD subcodes [16]. SPDR is considered to have nearly complete national coverage of dispensed prescriptions [17]. Demographic and socioeconomic information was gathered from the Swedish Total Population Register (TPR) and the Longitudinal Integration Database for Health Insurance and Labour Market Studies (LISA; maintained by Statistics Sweden; annually updated information on education, income, employment status, and social benefits for all individuals aged ≥ 16 years residing in Sweden [18]). Demographic characteristics, including sex, date of birth, migration, and cohabitation status, were obtained from the TPR, which covers the entire Swedish population and is widely used in epidemiological research [19].

The study is reported in accordance with STROBE guidelines.

### Population sample

The baseline study population consists of all individuals aged 25–80 years who were residing in Sweden on December 31, 2010. From this group, individuals who died or emigrated during the study period, those with pathological fractures or previous nerve injuries, and those with missing information on country of birth, fracture codes, prior medication use, or medication doses, were excluded (see Figure). Thus, osteoporosis-related pathological fractures were excluded to avoid confounding by such osteoporosis-related pain trajectories and medication use. Previous medication overuse (see below) before fracture-nerve injury diagnosis/surgery was also excluded (see Figure). The final study sample of this nationwide register-based cohort study comprised approximately 5.8 million individuals (see Figure).

**Figure:**
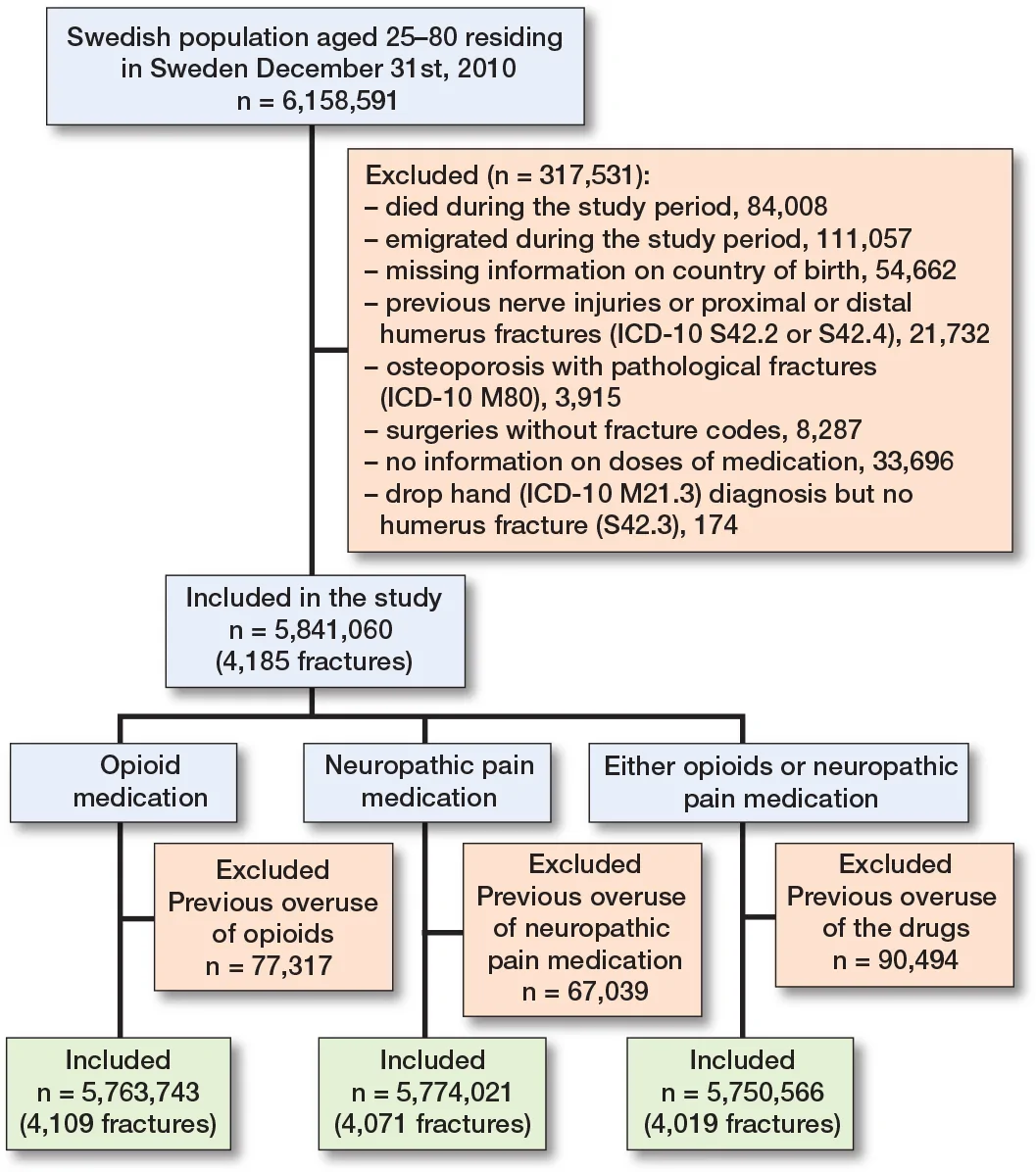
Flowchart of extracted data of total numbers of individuals with exclusions and the number of nonoperatively or surgically treated diaphyseal humerus fractures with and without nerve injury from a Swedish population stratified by medication.

### Assessment of variables

Individuals with diaphyseal humerus fractures, treatment strategies (information concerning secondary surgery for non-union or infection not possible to achieve), and nerve injuries in the Swedish National Patient Register (NPR) using the International Classification of Diseases, 10th Revision, Swedish Clinical Modification (ICD-10-SE) codes and the Nordic Classification of Surgical Procedures (NCSP), version 1.16 (see Supplementary file 1; surgical code for closed reduction included in the surgically treated group), during the period 2007–2015, were identified [16,20]. To ensure data integrity and avoid overcounting, the first chronological occurrence of a fracture was identified for each individual during the study period by the presence of either of the 2 ICD codes. Any subsequent records for the same individual were considered duplicates or follow-up entries and were excluded. Therefore, each individual contributed only once to the analysis, representing their index fracture event.

Regarding laterality and refractures, our analysis focused on the individual (patient) level rather than individual bone events. As only the first recorded fracture during the study period was included, subsequent fractures (whether on the same or contralateral side) were not analyzed. By grouping both ICD codes into a single “fracture” category, the clinical diagnosis was prioritized over anatomical laterality to streamline comparisons between exposure groups (i.e., no cases in our cohort in which the first event during the study period presented with both ICD codes at the same time).

Exposure groups were constructed as 5 mutually exclusive categories based on a clinical hierarchy to ensure no patient was counted in more than 1 group:

- a population reference group consisting of the final study sample (excluding groups b–e), i.e., individuals with no recorded fracture or nerve injury codes during the study period;
- nonoperatively treated diaphyseal humerus fractures, i.e., individuals with a fracture code but no surgical codes or nerve injury diagnosis;
- surgically treated diaphyseal humerus fractures, i.e., individuals who underwent surgical treatment for the fracture but had no associated nerve injury;
- nonoperatively treated diaphyseal humerus fractures with nerve injury, i.e., individuals with a fracture and a nerve injury diagnosis, but no surgical fracture intervention;
- surgically treated diaphyseal humerus fractures with nerve injury, i.e., individuals who underwent surgery for the fracture and had an associated nerve injury.

Nerve injury, with surgery when applicable, was defined using the ICD-10 diagnostic codes with NOMESCO codes as defined in Supplementary file 1 and considered present if recorded within 3 months of the fracture diagnosis. Individuals with a registered nerve injury in the year preceding the fracture were excluded to reduce the risk of misclassification of pre-existing conditions.

Prescriptions for opioids and medications often used for neuropathic pain were identified using relevant Anatomical Therapeutic Chemical (ATC) codes from the SPDR (see Supplementary file 1). To ensure focus on treatments started after the fracture and to minimize capturing acute peri-traumatic prescriptions, a 2-month washout period following the fracture diagnosis was implemented, during which dispensations were not included.

### Outcome definition

The outcomes were high (sustained) use of pain medication during the first and second years after the fracture, assessed as 3 separate binary outcomes: (i) high use of opioids, (ii) high use of neuropathic pain medication, and (iii) high combined use, defined as high use of opioids and/or neuropathic pain medication in the same follow-up year. For each follow-up year, medication exposure was measured by the proportion of days covered (PDC), derived from Defined Daily Doses (DDDs). This was calculated by dividing the number of days covered by the medication supply by the number of days in the follow-up period. PDC values were then dichotomized at a threshold of 0.60 (60%), with individuals showing ≥ 60% coverage categorized as having high medication use, indicating sustained use rather than short-term or intermittent treatment. The same PDC-based definition and threshold were applied to all 3 outcomes. Demographic and socioeconomic characteristics (age, sex, country of birth, income, and cohabitation status), defined in the section “Demographic and socioeconomic variables,” were examined for their associations with each outcome and were included in the adjusted models.

### Demographic and socioeconomic variables

Demographic and socioeconomic characteristics were obtained from the Total Population Register (TPR) and the Longitudinal Integrated Database for Health Insurance and Labour Market Studies (LISA). Age was measured at the time of fracture (or corresponding index date for the population reference group) and categorized as indicated in Table 1. Sex was recorded as male or female. Country of birth was classified as Sweden-born or foreign-born. Disposable household income was measured in the year preceding the index date and categorized into tertiles based on the national distribution. Cohabitation status was defined as living with a partner or living alone at the index date.

**Table 1:** Descriptive characteristics of the study population with nonoperatively or surgically treated diaphyseal humerus fractures, with and without nerve injury, in Sweden. Values are number of individuals and proportion (%) within each group, representing absolute risks of high use of opioids and neuropathic pain medication during the specified follow-up periods, as well as demographic and socioeconomic characteristics

Factor	Humerus fractures				
	Reference population n = 5,836,875	Nonoperatively treated n = 3,129	Surgically treated n = 903	Nonoperatively treated with nerve injury n = 79	Surgically treated with nerve injury n = 74
Previous overuse					
Opioids	77,205 (1.3)	86 (2.8)	26 (2.9)	0 (0.0)	0 (0.0)
Neuropathic pain medication	66,925 (1.2)	79 (2.5)	29 (3.2)	2 (2.5)	4 (5.4)
Either opioids or neuropathic pain medication	90,328 (1.6)	113 (3.6)	46 (5.1)	2 (2.5)	5 (6.8)
1st year overuse					
Opioids	321,972 (5.5)	586 (19)	359 (40)	15 (19)	36 (49)
Neuropathic pain medication	176,562 (3.0)	195 (6.2)	81 (9.0)	5 (6.3)	13 (18)
Either opioids or neuropathic pain medication	452,695 (7.8)	698 (22)	376 (42)	16 (20)	40 (54)
2nd year overuse					
Opioids	28,412 (0.5)	56 (1.8)	27 (3.0)	4 (5.1)	2 (2.7)
Neuropathic pain medication	76,351 (1.3)	84 (2.7)	34 (3.8)	2 (2.5)	4 (5.4)
Either opioids or neuropathic pain medication	103,803 (1.8)	133 (4.2)	59 (6.5)	5 (6.3)	6 (8.1)
Sex					
Men	2,919,877 (50)	1,275 (41)	392 (43)	40 (51)	33 (45)
Women	2,916,998 (50)	1,854 (59)	511 (57)	39 (49)	41 (55)
Age group					
25–34	1,085,258 (19)	294 (9.4)	100 (11)	12 (15)	18 (24)
35–44	1,232,376 (21)	316 (10)	80 (8.9)	9 (11)	5 (6.8)
45–54	1,180,132 (20)	460 (15)	112 (12)	18 (23)	11 (15)
55–64	1,138,548 (20)	791 (25)	221 (24)	12 (15)	16 (22)
65–80	1,200,561 (21)	1,269 (41)	390 (43)	28 (35)	24 (32)
Income					
Low	1,416,018 (24)	765 (24)	207 (23)	26 (33)	14 (19)
Middle	2,028,138 (35)	1,108 (35)	316 (35)	26 (33)	28 (38)
High	2,392,719 (41)	1,256 (40)	380 (42)	27 (34)	32 (43)
Country of birth					
Native	4,904,720 (84)	2,691 (86)	798 (88)	69 (87)	65 (88)
Immigrant	932,155 (16)	438 (14)	105 (12)	10 (13)	9 (12)
Cohabiting status					
Living alone	2,355,708 (40)	1,600 (51)	486 (54)	41 (52)	41 (55)
Cohabiting	3,481,167 (60)	1,529 (49)	417 (46)	38 (48)	33 (45)

Individuals with previous overuse before the diagnosis–surgery of the fracture-nerve injuries are included in the table but were excluded (see Figure) before the analysis using the Cox proportional hazards regression models. However, the previous overuse during the first year was included as an adjustment variable in the regression model during the second year (see Table 2).

### Previous medication use

Previous high use of opioids and neuropathic pain medication was assessed during the year preceding the index date using the same PDC-based definition as for the outcome. Individuals with ≥ 60% coverage during the preceding year were classified as having previous high medication (over)use. These individuals were excluded prior to the analysis using Cox proportional hazards regression models (see Figure) but are presented in Table 1 for completeness. In addition, previous overuse during the first year after the diagnosis or diagnosis-surgery was included as an adjustment variable in the regression model during the second year to capture pre-existing patterns of sustained pharmacological treatment and underlying vulnerability to prolonged medication use.

### Statistics

Descriptive statistics were used to summarize demographic and clinical characteristics across exposure groups and are presented as counts and percentages. To estimate associations between fracture category, defined by treatment strategy and presence of nerve injury, and long-term use of opioids or neuropathic pain medication, we applied Cox proportional hazards regression models with a fixed follow-up time set to 1 for all individuals. Under this specification, the Cox model yields estimates equivalent to relative risks (RRs) for binary outcomes. This approach allows direct estimation of RRs and avoids the interpretational bias of odds ratios when outcomes are not rare as in our study. Robust standard errors were used to calculate 95% confidence intervals (CIs). Analyses were first performed without adjustment (crude RR) and then adjusted (aRR) for age, sex, country of birth, income, and cohabitation status, and for previous high use of pain medication during the first year when analyzing the second year. The purpose of the adjustment was to estimate fracture-related associations in medication use beyond demographic and socioeconomic differences in the population, rather than to support causal inference [21]. As the study included the entire eligible Swedish population, aged 25 to 80 years, no formal power calculation was performed. All analyses were conducted using Stata version 14.1 (StataCorp, College Station, TX, USA).

### Ethics, data sharing plan, funding, use of AI, and disclosures

After approval from the Regional Ethical Committee in South Sweden (#:2014-856) and consent from the data safety committees, the Swedish authorities anonymized the data before exporting it.

The detailed individual data of the subjects or the database is not publicly available for ethical and/or legal reasons due to compromising patient privacy because the Ethical Committee have imposed these restrictions.

This work was supported by grants from Region Östergötland, Sweden (register number RÖ978765, RÖ985138, and RÖ1005512), the Swedish Research Foundation (grant number 2021-01942), Lund University, funds from Skåne University Hospital (2022-974), Elly Olsson’s Foundation for Medical Research, the Stig and Ragna Gorthon Foundation, and by ALF Grants in Region Skåne (register number 2022-Projekt0129).

Grammarly was used to improve the language and readability of the manuscript.

The authors have no conflicts of interests related to the present project. Complete disclosure of interest forms according to ICMJE are available on the article page, doi: 10.2340/17453674.2026.46808

## Results

### Study population and fracture characteristics

During 2007–2015, a total of 4,185 individuals were diagnosed with a diaphyseal humerus fracture (Figure). Of these, 977 (23%) underwent surgical treatment. Within the surgical treatment group, closed reductions (code NBJ09) accounted for 26% (n = 254) of the procedures (i.e., 5%, n = 13, among the surgically treated diaphyseal humerus fractures with nerve injury), while the remainder consisted of open reductions and other internal fixation methods.

Nerve injuries occurred concurrently in 153 individuals (3.7%), comprising 79 cases in nonoperatively managed fractures and 74 in surgically managed ones. In the group with the surgically treated fractures with nerve injury, 27 out of 32 nerve procedures (84%) were related to the radial nerve. Open fractures were uncommon (1.9%; Table 1).

Fractures were more prevalent in women (58%) than men (42%), with incidence rising with age and peaking in the 65–80-year group. Individuals with fractures, particularly when nerve injury was present, were more likely to live alone. Additionally, those who underwent nonoperative treatment for humerus fractures with nerve injury had lower incomes than the reference population. Demographic and socioeconomic distributions for each exposure group are detailed in Table 1. This table shows absolute risks as percentages (%) with corresponding counts (n) for high opioid and neuropathic pain medication use within each group. Specifically, the percentages indicate the proportion of individuals who met the high medication-use criteria during the follow-up period. These absolute risks provide a direct, clinically interpretable measure of medication use and serve as the basis for the relative risk estimates in the regression analyses (see below).

### Outcomes

#### Associations with demographic and socioeconomic characteristics

Beyond fracture category, several demographic and socioeconomic characteristics were associated with high pain medication use (Table 2). In the first year, high opioid use was more common among women (aRR 1.2, CI 1.2–1.2), older individuals (65–80 years: aRR 4.1, CI 4.1–4.2 vs 25–34 years), those with low income (aRR 1.5, CI 1.5–1.5 vs high income), and those living alone (aRR 1.2, CI 1.2–1.2). Similar associations were observed for neuropathic pain medication use, with a strong association seen for female sex (aRR 1.8, CI 1.8–1.9). These associations attenuated in the second year.

**Table 2:** Results from the Cox proportional hazards regression models used to calculate use of opioids and neuropathic pain medication in a Swedish population with nonoperatively or surgically treated diaphyseal humerus fractures with and without nerve injury presented as crude risk ratio or adjusted relative risk ratio (aRR)

	1st year		2nd year	
	Crude RR (CI)	aRR ^a^ (CI)	Crude RR (CI)	aRR ^a^ (CI)
**Opioid medication**				
Individuals				
Reference population	Reference	Reference	Reference	Reference
Humerus fracture treament				
Nonoperative	4.0 (3.7–4.4)	3.1 (2.8–3.4)	1.5 (1.1–2.2)	1.5 (1.0–2.1)
Surgical	11.6 (10.1–13.2)	9.5 (8.2–10.9)	1.2 (0.7–2.0)	1.2 (0.7–2.0)
Nonoperative with nerve injury	4.4 (2.5–7.6)	3.7 (2.1–6.6)	9.1 (2.9–28.2)	8.3 (2.6–26.1)
Surgical with nerve injury	17.6 (11.2–27.8)	17.6 (11.0–28.3)	1.5 (0.4–6.3)	1.6 (0.4–6.9)
Previous overuse				
No			Reference	Reference
Yes			221 (207–236)	208 (194–222)
Sex				
Men		Reference		Reference
Women		1.2 (1.2–1.2)		1.0 (0.9–1.0)
Age group				
25–34		Reference		Reference
35–44		1.6 (1.6–1.6)		1.5 (1.4–1.6)
45–54		2.2 (2.2–2.2)		1.6 (1.5–1.7)
55–64		3.0 (3.0–3.1)		1.7 (1.6–1.8)
65–80		4.1 (4.1–4.2)		1.4 (1.3–1.5)
Income				
Low		1.5 (1.5–1.5)		1.9 (1.8–2.0)
Middle		1.3 (1.3–1.3)		1.5 (1.4–1.6)
High		Reference		Reference
Country of birth				
Native		Reference		Reference
Immigrant		1.1 (1.1–1.1)		0.9 (0.9–1.0)
Cohabiting				
Yes		Reference		Reference
Living alone		1.2 (1.2–1.2)		1.3 (1.2–1.3)
**Neuropathic pain medication**				
Individuals				
Reference population	Reference	Reference	Reference	Reference
Humerus fracture treament				
Nonoperative	2.1 (1.7–2.5)	1.7 (1.4–2.0)	1.2 (0.8–1.7)	1.3 (0.9–1.9)
Surgical	3.2 (2.4–4.2)	2.7 (2.0–3.5)	1.6 (1.0–2.8)	1.7 (1.0–3.0)
Nonoperative with nerve injury	2.7 (1.0–7.4)	2.4 (0.9–6.5)	1.2 (0.1–10.2)	1.2 (0.1–10.5)
Surgical with nerve injury	7.2 (3.6–14.6)	6.8 (3.4–13.8)	0.5 (0.1–3.9)	0.5 (0.1–4.2)
Previous overuse				
No			Reference	Reference
Yes			160 (156–165)	170 (165–174)
Sex				
Men		Reference		Reference
Women		1.8 (1.8–1.9)		1.0 (1.0–1.1)
Age (years)				
25–34		Reference		Reference
35–44		1.6 (1.6–1.7)		1.0 (0.9–1.0)
45–54		2.1 (2.0–2.1)		0.8 (0.8–0.8)
55–64		2.3 (2.3–2.4)		0.7 (0.6–0.7)
65–80		2.4 (2.4–2.5)		0.5 (0.4–0.5)
Income				
Low		1.7 (1.6–1.7)		1.1 (1.1–1.2)
Middle		1.3 (1.3–1.4)		1.1 (1.1–1.1)
High		Reference		Reference
Country of birth (COB)				
Native		Reference		Reference
Immigrant		1.1 (1.1–1.1)		0.7 (0.6–0.7)
Cohabiting				
Yes		Reference		Reference
Living alone		1.3 (1.3–1.3)		1.2 (1.2–1.2)

a Risk ratio (RR) adjusted for age, sex, country of birth (COB), income, and cohabiting, as well as for previous medication overuse (during the first year) regarding the second year.

CI = 95% confidence interval.

#### Use of opioids

In the first year after fracture, all fracture categories showed substantially higher risks of high opioid use compared with the reference population (see Table 2). The highest adjusted relative risks were observed among surgically treated fractures with nerve injury (aRR 17.6, CI 11.0–28.3), followed by surgically treated fractures without nerve injury (aRR 9.5, CI 8.2–10.9). Nonoperatively treated fractures also showed elevated risks (aRR 3.1, CI 2.8–3.4), with higher risks when a nerve injury was present (aRR 3.7, CI 2.1–6.6).

In the second year, adjusted RRs declined across most fracture categories but remained elevated relative to the reference population. Notably, nonoperatively treated fractures with nerve injury continued to show a high risk for opioid use (aRR 8.3, CI 2.6–26.1), whereas the risk among nonoperatively treated fractures without nerve injury attenuated further (aRR 1.5, CI 1.0–2.1) (see Table 2).

Adjustment for demographic and socioeconomic variables reduced the magnitude of RRs but did not alter the overall pattern of associations across fracture categories (see Table 2).

#### Use of neuropathic pain medication

During the first year, increased use of neuropathic pain medication was observed in all fracture categories except nonoperatively treated fractures with nerve injury (aRR 2.4, CI 0.9-6.5) (Table 2). The highest risk was seen among surgically treated fractures with nerve injury (aRR 6.8, CI 3.4–13.8).

In the second year, no fracture category showed a statistically significant increase in neuropathic pain medication use compared with the reference population (see Table 2).

#### Combined use of opioids or neuropathic pain medication

Analysis of combined high use of either opioids or neuropathic pain medication showed patterns similar to those observed for neuropathic pain medication alone, with elevated risks primarily confined to the first year after fracture (see Table 1; Supplementary Table 1).

#### Medication types

Among opioid prescriptions, tramadol and tapentadol (N02AX) were most frequently dispensed, followed by oxycodone (N02AA05). For neuropathic pain medication, venlafaxine (N06AX16) was the most prescribed agent (Table 3).

**Table 3:** Descriptive data of the total ATC codes used and count and proportion (%) of prescriptions for each ATC code regarding opioids and neuropathic pain medication among individuals with nonoperatively and surgically treated diaphyseal humerus fractures with and without nerve injury

Category ATC code	Generic name	Prescriptions n (%)
Opioids (57%)		
N02AA01	Morphine	256,974 (5.7)
N02AA05	Oxycodone	531,264 (12)
N02AA55	Oxycodone and naloxone	21,955 (0.5)
N02AE01	Buprenorphine	171,475 (3.8)
N02AG02	Ketobemidone and spasmolytics	7,643 (0.2)
N02AB03	Fentanyl	59,810 (1.3)
N02AJ	Codeine combined with non-opioid analgesics	505 (0.01)
N02AX	Other opioids (tramadol and tapentadol)	1,512,616 (34)
R05DA04	Codeine	24,219 (0.5)
Neuropathic pain medication (43%)		
N03AX12	Gabapentin	237,020 (5.3)
N03AX16	Pregabalin	463,268 (10)
N06AA09	Amitriptyline	418,636 (9.3)
N06AX16	Venlafaxine	521,038 (12)
N06AX21	Duloxetine	281,281 (6.2)

## Discussion

We aimed to study pain medication use after diaphyseal humerus fractures, focusing on nerve injury. We found that long-term use of pain medication was more common than in the general population, particularly during the first year after injury. The relative risks for long-term use of pain medication were high among the groups, particularly in surgically treated fractures involving mainly radial nerve injuries, despite the low absolute numbers of concomitant nerve injuries indicated by broader confidence intervals. In the second year, individuals with nonoperatively treated diaphyseal humerus fractures with a nerve injury showed a higher risk of prolonged opioid use, with risks surpassing those of other fracture groups. Additionally, a greater risk of pain medication usage was noted among women, older adults, lower-income individuals, and those living alone. This suggests that fracture-related factors add to existing demographic and socioeconomic patterns in pain medication consumption.

The overall proportion of recorded nerve injuries was 3.7%, substantially lower than the approximately 12% reported in clinical series and systematic reviews for radial nerve injury [12]. Lower incidences of about 8% have also been reported elsewhere, particularly in settings dominated by low-energy trauma in older individuals [1-3]. In our data, nerve injuries were identified when diagnosed at the time of fracture or within 3 months thereafter; however, under-recording is possible. Radial nerve injury after a diaphyseal humerus fracture is considered to have a favorable prognosis and is often transient [12,22]. Clinicians may be less likely to systematically code diagnoses, which could contribute to under-ascertainment in register-based data [16,20].

Nonoperative treatment remains the standard of care for diaphyseal humerus fractures, despite a higher risk (11–15%) of non-union compared with surgically treated fractures (3–6%) [6,20-23], with apparently no difference in persistent radial nerve palsy between treatment strategies [11]. In our material, 23% of fractures were surgically treated within the first 6 months, consistent with data from the Swedish Fracture Register, which indicates stable treatment patterns over the past decade [23]. Against this clinical background, our findings suggest that surgical treatment is associated with an early but largely transient increase in opioid use, whereas nonoperatively treated fractures with nerve injury show a more persistent elevation in opioid use through the second year.

We applied a 2-month washout period to focus on post-fracture pharmacological treatment, assuming this period reflects typical acute pain management following a humerus fracture [24]. The observed early increase in opioid use after surgery, which also reflects the fracture locations [25], is consistent with previous reports of elevated postoperative opioid use among both opioid-naive and previously exposed individuals [26-28]. The decrease in opioid-related risks among surgically treated groups during the second year contrasts with the persistent high risk in nonoperatively managed fractures with nerve injury. Moreover, previous opioid use strongly predicted continued use into the second year, emphasizing the importance of early prescribing practices.

The risk of high use of neuropathic pain medication was considerably lower than that observed for opioids. Neuropathic agents were used more frequently in the first year, particularly among surgically treated fractures with nerve injury, but no fracture group showed a significantly increased risk in the second year. This pattern suggests that persistent neuropathic pain may be less prevalent than prolonged nociceptive or mixed pain related to fracture healing and surgical intervention. In contrast, the risk of opioid use in the second year disappeared among surgically treated fractures, whereas it remained elevated among individuals with nonoperatively treated fractures and concomitant nerve injury, a pattern that is clinically plausible given the potential for persistent pain. In addition, individuals with opioid use in the first year had a substantially increased risk of continued use in the second year. These findings emphasize the importance of adopting multimodal pain management approaches that combine pharmacological and non-pharmacological methods. This includes the appropriate use of non-opioid analgesics, neuropathic pain medications, when necessary, structured follow-up, and coordination between orthopedic and primary care providers. Such strategies are especially relevant for individuals with prolonged pain medication use across various healthcare settings, where prescribing practices and patient–clinician interactions may differ. This is particularly relevant for those at higher risk of ongoing pharmacological treatment, as outlined in several guidelines [29,30]. However, given the low overall proportion of diagnosed nerve injuries, which were reflected in broad confidence intervals, these findings should be interpreted cautiously. Prescription patterns observed in our data may also reflect broader temporal trends, including the decline in tramadol prescribing and the concurrent increase in oxycodone use during the study period [31-32]. There may be a transition in treatment from orthopedic specialist care to primary care for humeral shaft fractures over time and, thus, the prescribing responsibility may shift. However, prescriber identity cannot be distinguished in the register data.

### Limitations

We used register-based definitions of medication dispensing as a proxy for pain and treatment burden. However, we lacked access to clinical data, including pain severity scores, neurological recovery, fracture-healing status, functional outcomes, and secondary surgery for non-union or infection. Although our outcome definitions were based on dispensed prescriptions and DDDs, we cannot confirm actual medication adherence or consumption. Furthermore, there is potential for misclassification of nerve injuries, particularly if diagnoses were not consistently recorded or coded, including the timing of the nerve injury relative to surgery (e.g., primary trauma-related vs secondary iatrogenic nerve injury). As a result, the nerve injury groups, with a lower absolute number of cases, are likely to comprise clinically heterogeneous conditions. We also did not directly model changes in medication use over time within individuals, and the relative risks for the first and second years were based on separate comparisons with the population reference group rather than from longitudinal within-individual contrasts. In addition, although we adjusted for key demographic and socioeconomic factors, residual confounding from unmeasured variables, such as comorbidities, psychological factors, or access to health care, cannot be ruled out. This may weaken subgroup-specific associations and should be considered when interpreting the findings. Although there is clinical variability within the surgically treated group, with 26% of cases managed with closed reduction, it is unlikely that this significantly affects the overall trends. Since the study is observational, it cannot establish causality beyond what the analyses suggest.

### Conclusion

Our nationwide register-based study found an increased risk of pain medication use after diaphyseal humerus fractures, especially opioid use in surgically treated fractures in the first year post-fracture and in nonoperatively treated fractures with nerve injuries in the second year. A greater risk of medication use was noted among women, older adults, lower-income individuals, and those living alone.

*In perspective*, these findings emphasize the importance of careful early pain management and follow-up of patients at increased risk, who are more likely to require prolonged medication use.

### Supplementary data

Supplementary Table and diagnostic and surgical codes used are available as supplementary data on the article home page, doi: 10.2340/17453674.2026.46808

## Supplementary Material



## Notes

EN, LBD: conceptualization; RP, JM: methodology and data curation; RP: formal analysis; EN, SA, RP, MZ, JM, LBD: data interpretation; EN, LBD: resources; EN, SA: writing original draft; EN, SA, RP, MZ, JM, LBD: revision of draft; all authors have read and agreed to the final version of the manuscript

The authors would like to thank Tina Folker for her administrative support.

Handling co-editors: Jeppe Vejlgaard Rasmussen and Philippe Wagner

*Acta* thanks Dennis Karimi and Tuomas Lähdeoja for help with peer review of this manuscript.
