# Supplementary material for: Long-term use of opioids and neuropathic medication after diaphyseal humerus fractures with or without nerve injury: a nationwide register-based cohort study of 4,185 patients
Source: Acta Orthop. 2026 Oct 30;97:790–8. doi: 10.2340/17453674.2026.46808 (PMC13721846; doi:10.2340/17453674.2026.46808)
Supplement: Supplementary file 1 [file ActaO-97-46808-s1.pdf]

Supplementary Data

Supplementary Table. Results from the Cox proportional hazards regression models used to calculate the use of either opioids or neuropathic pain medication in a Swedish population with nonoperatively or surgically treated diaphyseal humerus fractures with and without nerve injury presented as relative risk ratio (RR) and 95% confidence interval (CI)

		1st year		2nd year	
		RR (CI)	aRR (CI) [a]	RR (CI)	aRR (CI) [a]
**Either opioids or neuropathic pain medication**					
**Individuals**	General population	Reference	Reference	Reference	Reference
	Nonoperatively treated fractures	3.6 (3.3–3.9)	2.8 (2.6–3.1)	0.99 (0.75–1.3)	1.04 (0.79–1.4)
	Surgically treated fractures	9.2 (8.0–10.5)	7.6 (6.6–8.7)	0.78 (0.51–1.2)	0.81 (0.54–1.2)
	Nonoperatively treated fractures with nerve injury	3.5 (2.0–6.2)	3.0 (1.7–5.4)	3.3 (1.1–10.1)	3.3 (1.1–10.1)
	Surgically treated fractures with nerve injury	15.1 (9.4–24.2)	15.0 (9.2–24.4)	0.61 (0.15–2.5)	0.60 (0.14–2.5)
**Previous overuse**	No			Reference	Reference
	Yes			65.9 (64.3–67.5)	69.5 (67.8–71.3)
**Sex**	Men		Reference		Reference
	Women		1.3 (1.3–1.3)		1.2 (1.2–1.2)
**Age groups**	25–34		Reference		Reference
	35–44		1.5 (1.5–1.5)		1.0 (1.0–1.1)
	45–54		2.1 (2.1–2.1)		0.91 (0.88–0.94)
	55–64		2.8 (2.8–2.8)		0.76 (0.73–0.79)
	65–80		3.5 (3.5–3.6)		0.57 (0.55–0.59)
**Income**	Low		1.5 (1.5–1.56)		1.4 (1.3–1.4)
	Middle		1.3 (1.3–1.32		1.2 (1.2–1.2)
	High		Reference		Reference
**Country of birth (COB)**	Native		Reference		Reference
	Immigrant		1.1 (1.1–1.1)		0.74 (0.72–0.76)
**Cohabiting**	Yes		Reference		Reference
	Living alone		1.2 (1.2–1.2)		1.3 (12–1.3)

[a] Adjusted for age, sex, country of birth (COB), income, and cohabiting, as well as for previous medication overuse (during the first year) regarding the second year.

Supplementary File

A. General population = no humerus fracture and no nerve injury

Excluded:
Other humerus fractures
S42.2 Proximal humerus fractures
S42.4 Distal humerus fractures

Surgery code
NBJ09 Closed reduction
NBJ19 Open reduction
NBJ69 Plate and screw fixation
NBJ59 Medullary nail
NBJ29 External fixation
NBJ49 Wires, pins, or other
NBJ79 Screws

Nerve injury
S44.0 Injury of ulnar nerve at upper arm level
S54.0 Injury of ulnar nerve at forearm level
S44.1 Injury of median nerve at upper arm level
S54.1 Injury of median nerve at forearm level
S44.2 Injury of radial nerve at upper arm level
S54.2 Injury of radial nerve at forearm level
S44.3 Injury of axillary nerve
S44.4 Injury of musculocutaneous nerve
G56.1 Other lesions of median nerve
G56.2 Lesion of ulnar nerve
G56.3 Lesion of radial nerve
M21.3 Drop hand

Nerve suture
ACB21 median nerve
ACB22 radial nerve
ACB23 ulnar nerve
ACB29 other nerve

Nerve graft
ACC21 median nerve
ACC22 radial nerve
ACC23 ulnar nerve
ACC29 other nerve

Nerve decompression
ACC51 median nerve
ACC52 radial nerve
ACC53 ulnar nerve

Nerve exploration
ACA11 median nerve
ACA12 radial nerve
ACA 13 ulnar nerve
ACA 19 other nerve

Nerve transposition
ACC43 ulnar nerve

Every individual can only be in one of the 4 categories below

B. Nonoperatively treated fractures (no fracture surgery at all and no nerve injury)

S42.30 Diaphyseal humerus fracture, closed
S42.31 Diaphyseal humerus fracture, open

C. Fractures surgically treated (no nerve injury)

S42.30 Diaphyseal humerus fracture, closed
S42.31 Diaphyseal humerus fracture, open
and
NBJ09 Closed reduction
NBJ19 Open reduction
NBJ 69 Plate and screw fixation
NBJ 59 Medullary nail
NBJ29 External fixation
NBJ49 Wires, pins, or other
NBJ79 Screws

D. Nonoperatively treated fractures (no surgery at all) ***and*** nerve injury (irrespective of surgery for nerve injury)

S42.30 Diaphyseal humerus fracture, closed
S42.31 Diaphyseal humerus fracture, open
and
S44.0 Injury of ulnar nerve at upper arm level
S54.0 Injury of ulnar nerve at forearm level
S44.1 Injury of median nerve at upper arm level
S54.1 Injury of median nerve at forearm level
S44.2 Injury of radial nerve at upper arm level
S54.2 Injury of radial nerve at forearm level
S44.3 Injury of axillary nerve
S44.4 Injury of musculocutaneous nerve
G56.1 Other lesions of median nerve
G56.2 Lesion of ulnar nerve
G56.3 Lesion of radial nerve
M21.3 Drop hand

with or without surgery for nerve injury

Nerve suture
ACB21 median nerve
ACB22 radial nerve
ACB23 ulnar nerve
ACB29 other nerve

Nerve graft
ACC21 median nerve
ACC22 radial nerve
ACC23 ulnar nerve
ACC29 Other nerve

Nerve decompression
ACC51 median nerve
ACC52 radial nerve
ACC53 ulnar nerve

Nerve exploration
ACA 11 median nerve
ACA12 radial nerve
ACA13 ulnar nerve
ACA19 other nerve

Nerve transposition
ACC43 ulnar nerve

E. Fractures surgically treated ***and*** nerve injury (irrespective of surgery for nerve injury)

S42.30 Diaphyseal humerus fracture, closed
S42.31 Diaphyseal humerus fracture, open

and

Fracture surgery
NBJ09 Closed reduction
NBJ19 Open reduction
NBJ 69 Plate and screw fixation
NBJ 59 Medullary nail
NBJ29 External fixation
NBJ49 Wires, pins, or other
NBJ79 Screws

Combined with
S44.0 Injury of ulnar nerve at upper arm level
S54.0 Injury of ulnar nerve at forearm level
S44.1 Injury of median nerve at upper arm level

S54.1 Injury of median nerve at forearm level
S44.2 Injury of radial nerve at upper arm level
S54.2 Injury of radial nerve at forearm level
S44.3 Injury of axillary nerve
S44.4 Injury of musculocutaneous nerve
G56.1 Lesion of median nerve
G56.2 Lesion of ulnar nerve
G56.3 Lesion of radial nerve
M21.3 Drop hand

With or without surgery for nerve injury
Nerve suture
ACB21 median nerve
ACB22 radial nerve
ACB23 ulnar nerve
ACB29 other nerve

Nerve graft
ACC21 median nerve
ACC22 radial nerve
ACC23 ulnar nerve
ACC29 other nerve

Nerve decompression
ACC51 median nerve
ACC52 radial
ACC53 ulnar nerve

Nerve exploration
ACA11 median nerve
ACA12 radial nerve
ACA13 ulnar nerve
ACA19 other nerve

Nerve transposition
ACC43 ulnar nerve

ATC codes

Opioids
N02AA01 morphine
N02AA05 oxycodone
N02AA55 oxycodone and naloxone
N02AE01 buprenorphine
N02AG02 ketobemidone and spasmolytics
N02AB03 fentanyl
N02AJ codeine, tramadol, and oxycodone in combination with non-opioid analgesics
N02AX tramadol and tapentadol
R05DA04 codeine

Neuropathic pain medication
N06AA09 amitriptyline
N06AX16 venlafaxine
N06AX21 duloxetine
N03AX16 pregabalin
N03AX12 gabapentin
